# Deep Learning for Fully-Automated Localization and Segmentation of Rectal Cancer on Multiparametric MR

**DOI:** 10.1038/s41598-017-05728-9

**Published:** 2017-07-13

**Authors:** Stefano Trebeschi, Joost J. M. van Griethuysen, Doenja M. J. Lambregts, Max J. Lahaye, Chintan Parmar, Frans C. H. Bakers, Nicky H. G. M. Peters, Regina G. H. Beets-Tan, Hugo J. W. L. Aerts

**Affiliations:** 1 Department of Radiology, the Netherlands Cancer Institute, Amsterdam, The Netherlands; 2 GROW School for Oncology and Developmental Biology, Maastricht University Medical Center, Maastricht, The Netherlands; 3 Department of Radiation Oncology and Radiology, Dana-Farber Cancer Institute, Brigham and Women’s Hospital, Harvard Medical School, Boston, USA; 4 Department of Radiology, Maastricht University Medical Centre, Maastricht, The Netherlands; 5 Department of Radiology, Zuyderland Medical Center, location Heerlen, Heerlen, The Netherlands

## Abstract

Multiparametric Magnetic Resonance Imaging (MRI) can provide detailed information of the physical characteristics of rectum tumours. Several investigations suggest that volumetric analyses on anatomical and functional MRI contain clinically valuable information. However, manual delineation of tumours is a time consuming procedure, as it requires a high level of expertise. Here, we evaluate deep learning methods for automatic localization and segmentation of rectal cancers on multiparametric MR imaging. MRI scans (1.5T, T2-weighted, and DWI) of 140 patients with locally advanced rectal cancer were included in our analysis, equally divided between discovery and validation datasets. Two expert radiologists segmented each tumor. A convolutional neural network (CNN) was trained on the multiparametric MRIs of the discovery set to classify each voxel into tumour or non-tumour. On the independent validation dataset, the CNN showed high segmentation accuracy for reader1 (Dice Similarity Coefficient (DSC = 0.68) and reader2 (DSC = 0.70). The area under the curve (AUC) of the resulting probability maps was very high for both readers, AUC = 0.99 (SD = 0.05). Our results demonstrate that deep learning can perform accurate localization and segmentation of rectal cancer in MR imaging in the majority of patients. Deep learning technologies have the potential to improve the speed and accuracy of MRI-based rectum segmentations.

## Introduction

Magnetic Resonance Imaging (MRI) is an integral part of the diagnostic work-up of rectal cancer and plays an important role in treatment planning. In addition, MRI can play a role in predicting clinically relevant endpoints, one of the most important ones being the response to neoadjuvant treatment^1–3^. Predicting which patients will show a very good response to treatment can have important clinical implications, since these patients may be considered for organ-preserving treatment strategies (local excision or watchful waiting) as an alternative to standard surgical resection^4^. In carefully selected patients these organ preserving treatments can considerably improve quality of life with a good oncological outcome.

A promising technique to assess response to neoadjuvant treatment is diffusion-weighted MRI (DWI). Various studies have shown that – as an addition to standard morphological MRI – DWI can aid in assessing response to chemoradiotherapy, in particular to differentiate residual tumour within areas of post-radiation fibrosis after CRT. For this purpose use of DWI is now even recommended in international clinical practice guidelines for rectal cancer imaging^4^.

Particularly good results have been shown for volumetric measurements derived from diffusion-weighted (DWI)^5–8^. Furthermore ADC and histogram features derived from DWI-MRI have shown promise as quantitative imaging biomarkers for therapeutic outcome^4,5,9,10^. Most of these measures are calculated from regions of interest (ROI) of the tumour that are typically obtained after manual tumour segmentation by experienced readers. Studies have indicated that whole-volume tumour segmentations, as opposed to single slice or sample measurements, provide the most reproducible and accurate estimates of the true tumour volumes^11,12^. The main problem with manual segmentation approaches, is that these are highly time consuming, up to 18 minutes per tumour^13^, and as such unlikely to be implemented into daily clinical practice. Previous studies have explored ways to automatically perform segmentations using software algorithms^6,13^. These approaches work best on diffusion-weighted images, as these highlight tumour and suppress background tissues, thereby providing a high tumour-to-background ratio.

**Figure 1:**
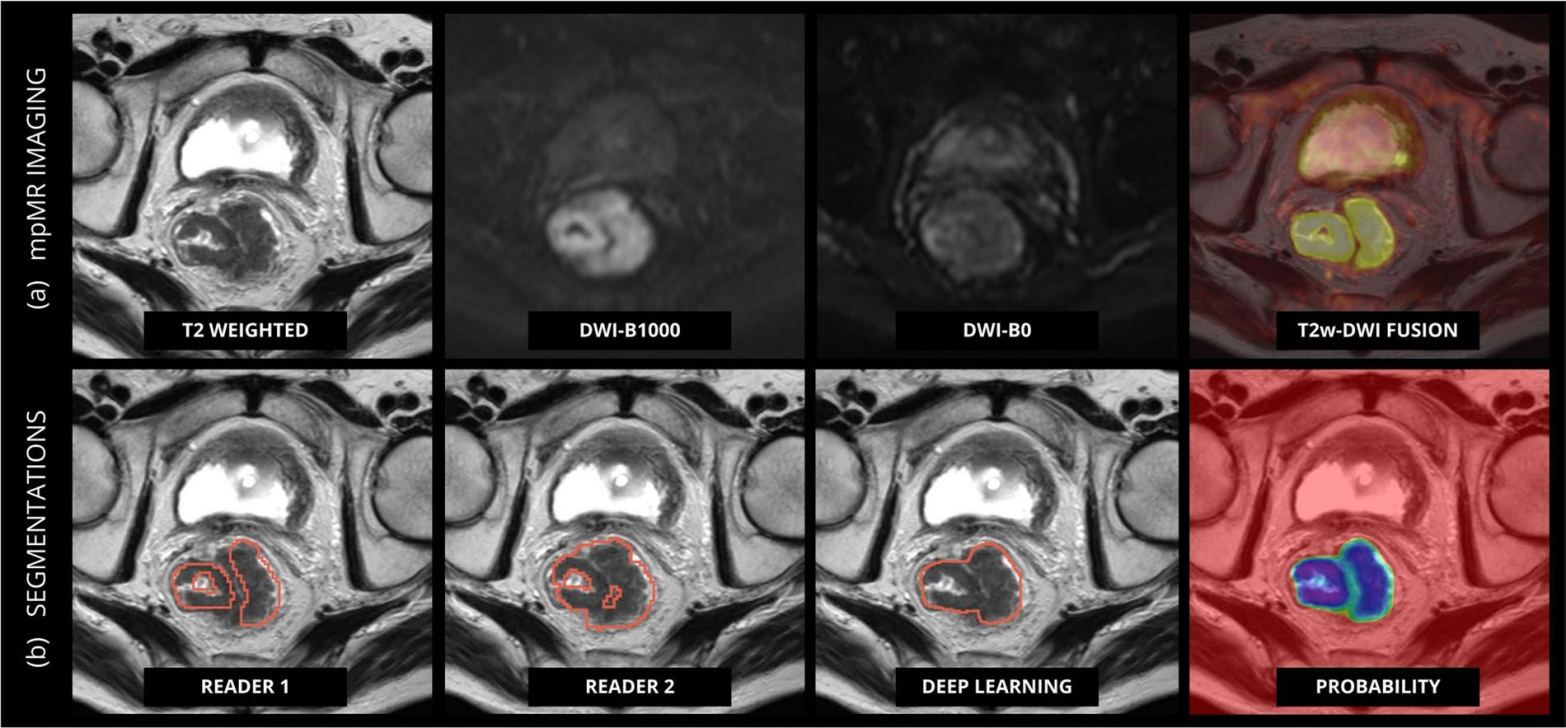
Example of Multiparametric MR in a rectal cancer patient. mpMR of the pelvis of a male patient with rectal cancer before the start of the treatment. Corresponding slices of different sequences on the transversal plane are shown. (**a**) The sequences are, in order: T2 weighted, DWI B1000, DWI B0 and fusion imaging between T2 weighted and the DWI B1000. Notice how anatomical structures and tissues surrounding the tumour – such as prostate, bladder, and seminal vesicles – and artefacts in general show the same hyper-intensity on the DWI of the tumour. (**b**) Delineations of the tumour done by (from the right left hand side): the experienced reader used for the training, the independent reader, the result of the algorithm and the corresponding probability map generated by the algorithm.

Unfortunately, high signal on DWI is not limited to tumour tissue only. Other anatomical structures in the pelvis (e.g. perirectal lymph nodes, prostate and ovaries) as well as artefacts may also show similar hyper-intensity and may not be recognized as such by typical simple segmentation algorithms causing these algorithms to fail to produce sufficiently accurate results^13^. In such cases, the manual input required from an experienced reader will not be limited to a threshold value or a seed point (in case of region growing), but will include manual corrections to adjust the segmentations for these effects^13^. Thus, there is an obvious need for smarter algorithms that can automatically localize and perform accurate segmentations of rectal tumours, which can reduce the need of expert input (Fig. 1).

Such fully automatic alternatives would also facilitate the generation of segmentations for large cohort studies, which is beneficial especially in light of new research developments such as Radiomics^14,15^, where complex tumour phenotypical characteristics are quantified and correlated to diagnostic or prognostic factors. The computation of these features requires input in the delineation of the region of interest to be described.

Artificial intelligence (AI) aims to mimic cognitive, labour intensive tasks via complex computational models trained on top of existing datasets. A computational model trained using the input from expert readers (radiologists) to automatically localize and segment rectal cancer in MR images, could represent a potential solution to this problem.

Novel AI technologies, such as deep learning models, have been exploited in recent years with impressive results. Convolutional neural networks (CNNs) based deep learning approaches can learn feature representations automatically from the training data. The multiple layers of the CNNs aim to process the imaging data with different levels of abstractions, enabling the machine to navigate and explore large datasets and discover complex structures and patterns that can be used for prediction^16^. The advancement of these techniques has been made possible by the availability of large imaging data and the accessibility of dedicated hardware devices such as graphical processing units (GPU)^15,16^. Particularly in the field of biomedical imaging, deep learning has been largely exploited for detection and segmentation purpose, where these methods are proven to systematically outperform traditional machine learning techniques^17,19^.

In this study, deep learning methods (CNNs) have been used to fully automatically localize and segment rectum tumours. To evaluate the performance of deep learning based segmentations, we compared them to manual segmentations of two independent expert radiologists. Deep learning technologies have the potential to improve the speed and accuracy of MRI-based rectum segmentations in clinical settings.

## Background Work

To the best of our knowledge, few investigations were conducted on the automatic localization and segmentation of rectal cancer. Irving *et al*.^20^ proposed an automatic segmentation procedure, based on DCE-MRI, where the authors accounted for the multidimensional nature of DCE signal through a modified version of the supervoxel algorithm corrected by a graphical model producing successful results. Although DCE-MRI tends to give a much clearer and less noisy signal compared to DWI, our method achieved comparable results to the one presented in this study. The most popular semi-automatic approach is region growing. Day *et al*.^21^ used region growing on FDG-PET on phantoms, leading to better results than thresholding of the standardized uptake value (SUV). In this case the intent of the authors was to optimize treatment planning. Region growing was also used by van Heeswijk *et al*.^13^, who concluded that it could represent a more convenient replacement for manual delineation in terms of time. Although the results showed a decrease in the amount of time required, manual input was still required and a DSC > 0.7 could only be achieved when the result of the region growing was adjusted by an experienced radiologist.

## Material and Methods

### Subjects and Study Dataset

For this study we retrospectively selected 140 patients (97 males, median age 67, range 43–87) with biopsy proven locally advanced rectal carcinoma (LARC) from a previously reported bi-institutional study cohort^5^. No significant difference in clinical parameters was observed between the two centres (see Table 1). All patients in this cohort have undergone multiparametric (mp) MRI, consisting of T2 weighted and diffusion weighted imaging (DWI), prior to standard chemo-radiotherapy treatment (CRT), using either an Intera (Achieva) or Ingenia scanner (Philips Healthcare, Best, The Netherlands) (centre A, 91 patients) or a Magnetom Avanto system (Siemens Healthcare, Forchheim, Germany) (centre B, 49 patients) with a phased array surface coil. Both T2w and DWI sequences were axially angled perpendicular to the tumour axis defined on a sagittal scan. The diffusion sequence was performed using b-values B0, B500, and B1000 (centre A) or B1100 (centre B). Patients did not receive bowel preparation. As described previously, all research was performed according to guidelines and regulations of The Netherlands^5^. In short, according to the Dutch law, retrospective studies are not subject to the Medical Research Involving Human Subjects Act and informed consent is not required^22^. Detailed parameters of the sequences are specified in Table 2.

**Table 1:** Patient Characteristics.

	Centre A	Centre B	Both Centres	p-value
N	91	49	140	—
Males/Females	66/25	31/18	97/43	*p = 0*.*498*, *χ*^*2*^ *test*
Age	66.6 ± 9.3	65.6 ± 9.8	66.2 ± 9.4	*p = 0*.*554*, *t-test*
Tumour Volume^α^	19.0 ± 22.3 cm^3^	23.8 ± 29.3 cm^3^	20.7 ± 25.0 cm^3^	*p = 0*.*321*, *t-test*

α according to the segmentation performed by the experienced reader. No significant difference has been found between the two centres.

**Table 2:** Sequence parameters of the diffusion-weighted imaging used during the study period.

	Centre A			Centre B	
Repetition Time	4004–4829	4971	4172–5241	5100	4300
Echo Time	70	70	68–70		79
Number of Slices	50	24	20–24	34	34
Slice Thickness (mm)	5	5	5	5	6
Slice Gap (mm)	0.5	0.5	0.5	0.5	0
In-Plane Resolution	2.50 × 3.11 (−3.18)	1.87 × 2.31	1.82 × 2.27	1.70 × 1.30	2.0 × 2.0
Echo train length	1	1	1	1	1
N. Signal Averages	4	5	5	6	6
b-values	0, (100), 500, 1000	0, 500, 1000	0, (25,50,100), 500, 1000	0, 500, 1000	0, 300, 1100
Fat Suppr. Tech.	STIR^δ^	SPIR^γ^/fatsat^α^	SPAIR^β^	SPIR^γ^/fatsat^α^	SPIR^γ^/fatsat^α^
Echo Planar Im.	53–55	55	61	148	150

^α^Fat Saturation, ^β^Spectral Attenuated Inversion Recovery, ^γ^Spectral Pre-saturation with Inversion Recovery, ^δ^Short T1 Inversion Recovery.

Whole-volume tumour segmentations were available for all patients and were done by an experienced reader (Reader 1, D.M.J.L.) on the highest b-value (B1000 or B1100) DWI, according to methods previously reported^13^, where the reader created an initial segmentation using a simple region growing algorithm and manually adjusted to fit the borders of the tumour. These segmentations were used as ground truth. Additionally, segmentations performed on the same dataset and in the same manner by an independent reader (Reader 2, M.J.L.) were retrieved. These segmentations were used as additional check.

Imaging data of 140 patients were included in our analysis. Patients were assigned to discovery or validation dataset depending on their identifier: even numbers were assigned to discovery dataset (N = 70), odd numbers to validation dataset (n = 70). For the discovery dataset, there were no errors within the imaging data and segmentations, and therefore 70 cases were used for training. For the validation dataset, three cases had to be excluded due to misalignment between DWI and T2 caused by an error in the DICOM metatags, one case where the DWI suffered from severe ghosting artefacts, and one case in which the segmentation file was corrupted. This resulted in 65 cases that could be used for validation.

### Pre-processing

All images underwent standardization of the intensities, namely the intensity distribution was set to have mean zero and standard deviation one. Deformable registration was applied using the elastix toolbox^23,24^ to compensate for the anatomical displacement of organs and tissues in different imaging sequences during the acquisition procedure. The DWI-B0 was used as reference image, since it visualizes anatomical structures like the T2w and, at the same time, is well aligned to the DWI-B1000. The deformation field was estimated via adaptive stochastic gradient descent^25^ minimizing the advanced mattes mutual information^26^. Transform bending energy^27^ was used as penalty measure to correct for anatomically unrealistic transformations. To properly simulate the small, local movements in the bowels, a dense sampling grid of 4 mm together with a strong weight on the penalty measure (1:20) was applied.

### Deep Learning (CNN) architecture

**Figure 2:**
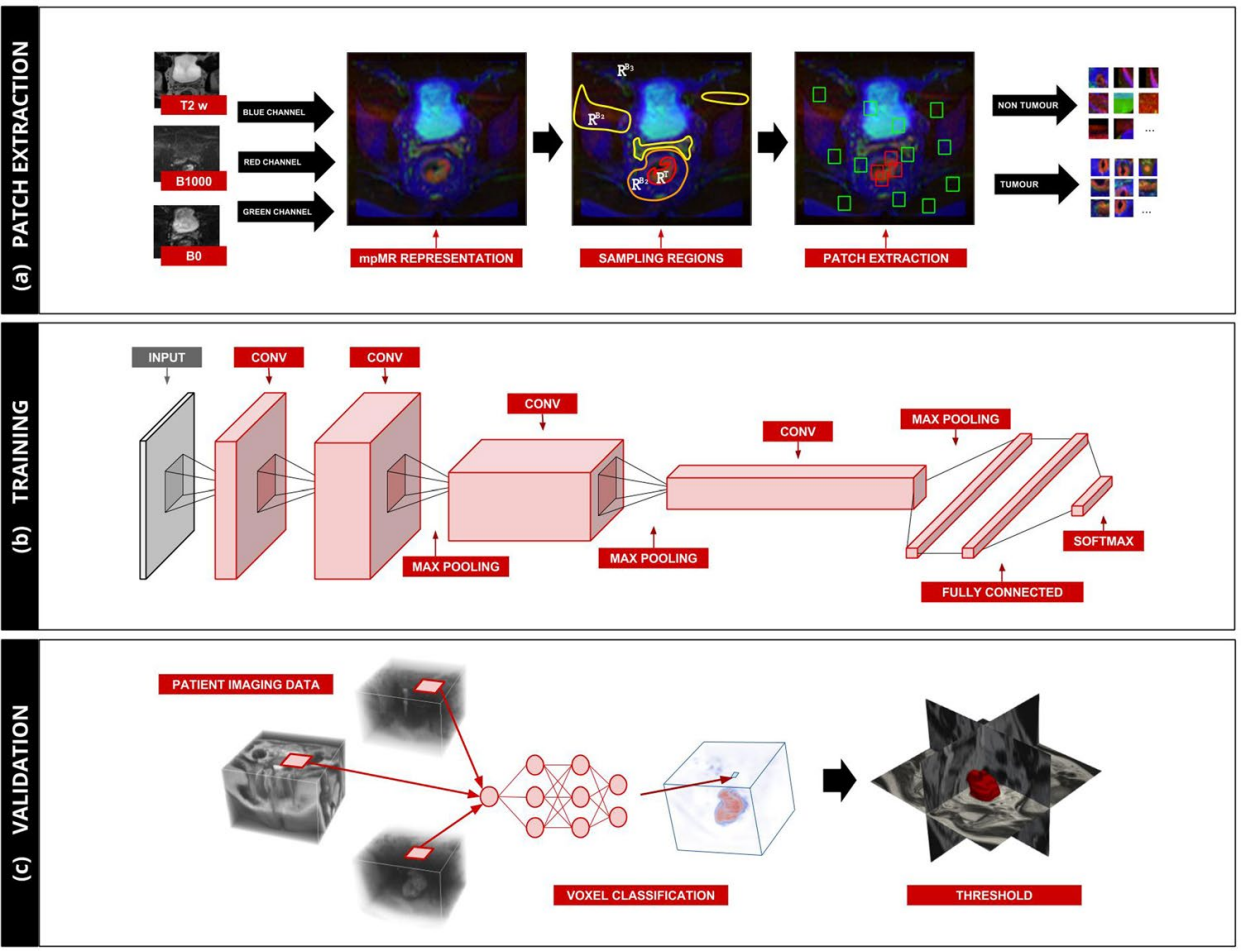
Scheme of the Proposed Solution. (**a**) On the left-hand side, a multiparametric representation of the imaging is created via fusion of corresponding slices from different sequences into the colour channels of the RGB model. In the centre, the label map is first divided in tumour region (R^T^) and the background regions (R^B^) according to the delineation done by the experienced reader. On the right-hand side, N voxels (together with their surrounding patch) are then randomly sampled from these regions to maintain a balance between number of voxels representing the tumour and number of voxels representing healthy tissue. (**b**) The architecture of the network, which is trained with the patches of the images in the discovery set. The patches of the images in the test set are used to control for model overfitting. (**c**) The 3D probability map is generated by classification of each voxel using the trained model. The probability map is thresholded to find the components where the probability of tumour is higher than the probability of healthy tissue. The largest component is selected as segmentation of the tumour.

In this study, a CNN architecture was implemented to function as voxel classifier. More specifically, for each voxel *v* we (1) extracted a fixed-size patch surrounding *v*, (2) classified the patch via a trained instance of the CNN, (3) collected the resulting probability, and (4) assigned the resulting probability to *v*. By repeating the procedure for each voxel of each image, we could generate a probability map, where *p*(*v*) is the probability of voxel *v* to represent tumour tissue. The segmentation was generated by thresholding of the probability maps (voxels with *p*(*v*) ≥ 0.5 were classified as “tumour”, and as “not tumour” otherwise) and subsequent selection of the largest connected component. Figure 2 offers a schematic synthesis of the whole process.

### Patch extraction

N voxels were randomly sampled from each of the foreground (i.e. tumour region) and background (i.e. non tumour region) regions. This ensured a balanced representation of the two classes during the training procedure. For each voxel, we extracted the surrounding in-plane patch of size M × M in all MR sequences. Each sequence was then fitted in one of the three channels of a standard RGB picture. The ground truth associated with each patch was the label of the central voxel.

Some regions are easier to classify, such as hypointensity on the DWI. Other regions instead are more challenging: for example, the prostate and the tumour regions have similar intensity and heterogeneity on T2w and DWI. Intuitively, the classifier should be able to spend more time learning to how to correctly classify these difficult regions of the image, and less time on regions that are easily classifiable. To translate this concept in implementation, the background class was divided in three regions: I) the area surrounding the tumour (R^B^_1_) defined by morphological dilation of the tumour segmentation with a spherical structural element of 1 cm radius; II) the regions hyper-intense on the DWI (R^B^_2_) defined by thresholding on the DWI at µ + 2σ. Since all images have been standardized, this operation will result on the thresholding at a value of 2.00; and III) the remaining areas (R^B^_3_) defined by the voxels not belonging to either R^B^_1_ or R^B^_2_. We sampled N/4 voxels from R^B^_1_ and R^B^_3_, and N/2 voxels from R^B^_2_, summing up to total N non-tumour voxels from the background class. Figure 2a shows a schematic representation of the sampling process.

### Network Definition

The network used for the classification of each patch is composed of a total of nine layers: two subsequent convolutional layers followed by a max pooling layer; a couple of smaller, subsequent convolutional layers, each followed by a max pooling layer; two fully connected layers at the end culminating in the output layer. This architecture is similar to the one proposed in ref.^28^, with the addition of a convolutional and max pooling layer. Dropout^29^ of 1/2 was used after each fully connected layers, and 1/3 after each max pooling layer^30^. Leaky rectified linear unit (ReLu)^31^ was used as a nonlinearity in each layer, except in the on the output layer, where a softmax was used instead. Small filters of 5 × 5 or 3 × 3 are used at each convolutional layer, along with stride one and full padding. Stride two was used in the max pooling. Twenty-four features were used in the first convolutional layer, number which doubles in each subsequent layer (i.e. 24, 48, 96, 192), amounting for a total of 360 filters throughout the entire network. Figure 2b shows a schematic representation the network structure. Cross entropy was used as cost function, together with a small L2 regularization on the network parameters. Adadelta^32^ with learning rate η = 0.001 and decay ρ = 0.9.

The discovery set was divided into training set (80%) and test set (20%). The training set was used for training the net, the test set was used alongside the training procedure to check for model overfitting. The training procedure was programmed to stop when no improvement on the cost δ of the test set was made for at least five consecutive epochs, where the improvement was defined as δ _EPOCH-1_ − δ _EPOCH_ > 10^–3^. The implementation of the algorithm was based on popular Python libraries: Lasagne and Theano^33^.

### Statistical Analysis

Segmentations were generated by feeding the patch of each voxel of mpMR to the algorithm and assigning the resulting probability to that voxel. The segmentation was generated by thresholding of the probability map at *p* = *0.5*. Additional selection of the largest component allowed the exclusion of small isolated voxels, which might pass unseen by most human readers. Figure 2c shows an example of this process.

In stochastic processes where samples are randomly selected, the result might often be not representative, with a significant variance in the final classification results. To evaluate the stability of the algorithm, we repeated the entire sampling, training and testing procedure four times and compared the segmentations generated by the different runs of the algorithm.

## Results

### Deep Learning (CNN) Training

**Figure 3:**
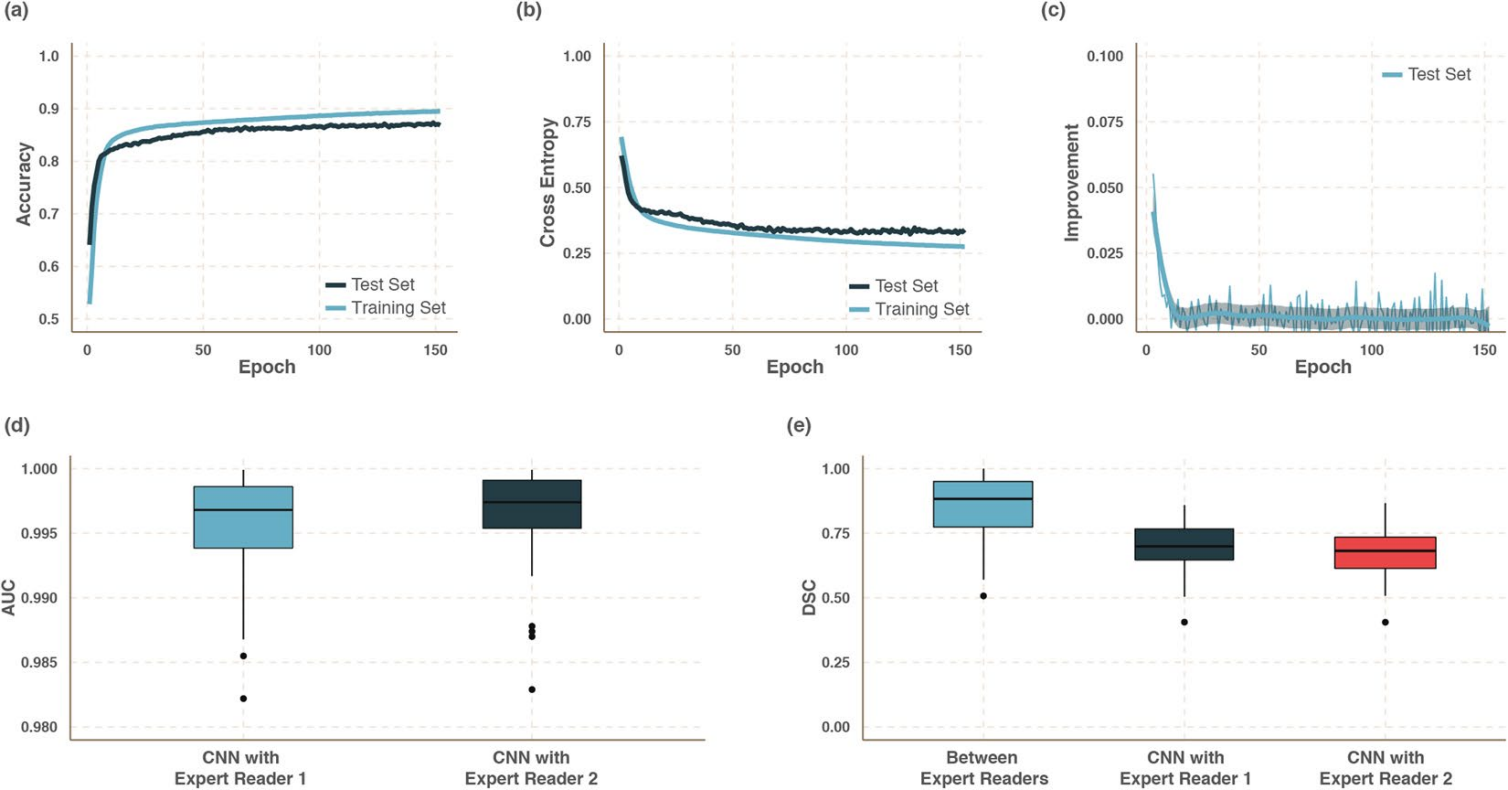
CNN Training and Validation. *Performance of the CNN on the discovery dataset:* (**a**) accuracy, (**b**) cross entropy and (**c**) improvement (Δ cross entropy). Improvement shown in panel (c), in computed on the test set only, preventing the model from overfitting. *Performance of the CNN on the validation dataset:* (**d**) the Area under the ROC curve (AUC) of the probability map with respect to the reader segmentation, and (**e**) Dice Similarity Coefficient (DSC) of the generated segmentations.

To develop a deep learning based algorithm for the fully automatic localization and segmentation of rectum tumours, we used independent discovery and validation datasets to develop an CNN-based network and validate its performance. The CNN was trained on multiparametric MR imaging (1.5T, T2-weighted and DWI) of 70 patients, using the segmentations performed by expert reader 1. For each patient, 5000 patches (size M × M = 21 × 21 voxels) were created by combining T2-weighted, and DWI images, for both tumour and non-tumour areas (Fig. 2a). The discovery data consisted of an independent discovery set (totalling 560 K patches) and test set (140 K patches). The algorithm reached a loss on the discovery set of 0.275 and 0.331 on the test set. The accuracy was 0.895 and 0.871, respectively. Figures 3a,b and c show the improvement of accuracy, the minimization of the cost function and its improvement over time. Notice from the graph presented in Fig. 3c that no major improvement on the cost function has been recorded after the 50th epoch.

### Validation of CNN classifier

The performance of the CNN classifier was validated on the validation dataset consisting of multiparametric MR imaging of 65 patients. For each patient volumetric tumour segmentations were generated and compared to both expert readers (Fig. 1b). Three cases had to be excluded where there was no agreement between expert readers. Therefore, data of 62 patients were used to validate the performance of the classifier. For all cases, the CNN could successfully generate volumetric tumour segmentations. To evaluate the performance of the CNN on a voxel-by-voxel bases, the area under the curve (AUC) was computed between the CNN probability maps and the segmentations of the experienced readers. The AUC of the resulting probability maps was very high for both readers, AUC = 0.99 (0.05 SD), with no significant difference between readers. Figure 3d shows AUC distributions for both readers.

From the probability maps we then generated volumetric segmentations. To evaluate the performance of the segmentation the *Dice Similarity Coefficient* (DSC) was used. The DSC is a statistical measure of spatial overlap frequently used to compare segmentations. The average DSC between the two expert readers was high (0.83, SD = 0.13). The DSC between the algorithm and Reader 1 was 0.68 (0.07 SD) and Reader 2 was 0.70 (0.07 SD), with no significant difference detected between the two distributions (*p* = *0*.*31*, *t-test*). Figure 3e shows DSC distributions between the algorithm and each reader, and between the two readers. Figure 1b show an example of a tumour successfully delineated by the algorithm (0.99 AUC, 0.85 DCS). Notice that the CNN was trained on segmentations of expert reader 1 in the discovery dataset. However, on the validation dataset the performance of the CNN was similar with both readers (Fig. 3e), demonstrating the generalizability of the network.

**Figure 4:**
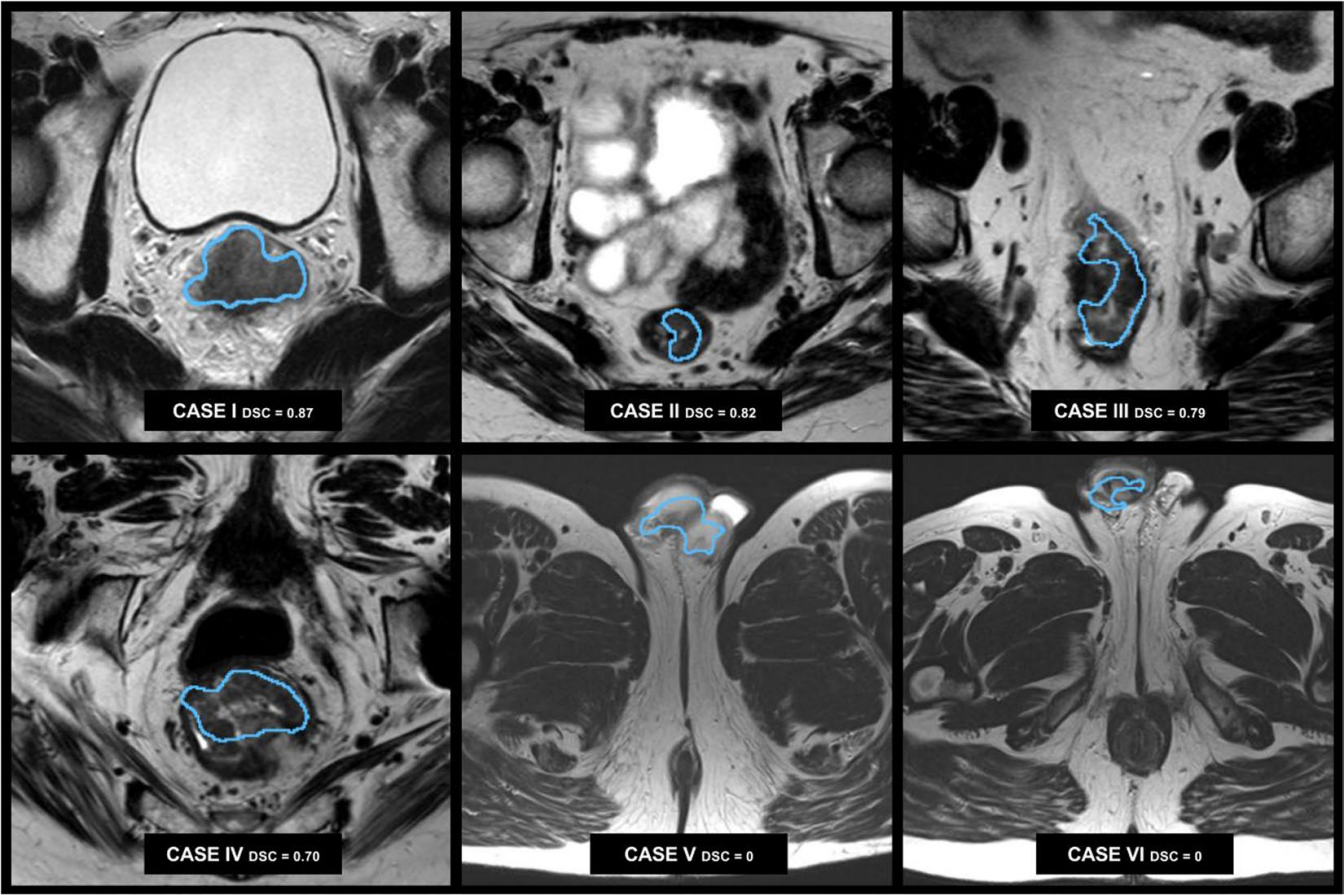
Example cases. Six example cases of the segmentation performed by the CNN. The algorithm correctly localized and segmented the tumour in case I to IV (small FOV images), but failed in cases with larger FOVs (cases V and VI) where parts of the cavernous bodies of the penis were erroneously included in these examples.

The algorithm resulted in a relatively poor result (DSC < 0.50) in ten cases. Figure 4 show an example of a tumour correctly classified by the algorithm but resulting in a poor DSC after thresholding and selection of the biggest connected component (0.98 AUC, 0 DCS), as the tumour was not the biggest component. In this case, the testicles (visualized in the lower part of the image) were assigned a probability greater than 0.5, enabling them to survive the threshold procedure and be selected as candidate segmentation.

### Stability of the Sampling Process

To access the stability of the sampling procedure and reproducibility of the model across different sampled voxels, the entire discovery and validation procedure was repeated additional four times. The final validation accuracy resulting from each individual training procedure (between 0.875 and 0.895), as well as the cross entropy (between 0.268 and 0.30), was stable. Each trained algorithm was used to generate the segmentations, resulting in four segmentations for each case. The *Intraclass Correlation Coefficient* (ICC) was used to access the agreement across different nets in terms of DSC. The overall agreement was very high (ICC = 0.83, 95% CI 0.77–0.88, *p* < 0.001).

## Discussion

Our aim was to develop a deep learning based network for the fully automatic localization and segmentation of locally advanced rectal tumours. Overall results show good performance of the algorithm, with segmentations comparable to those performed manually by an expert reader with a DSC of 0.70. In terms of classification, the high AUC of 0.99 suggests the ability of the algorithm to properly classify tumour voxels and therefore locate cancer tissue in the image. At visual inspection of the probability maps, one can appreciate how non-malignant hyperintensity of the DWI are attenuated with lower probabilities, whereas the tumour retains higher values.

After thresholding and selection of the largest component as candidate segmentation, the algorithm achieved an overall DSC of 0.70. At first glance, this is lower than the DSC reached between both readers on this dataset. In this case however the reader could rely on a semi-automatic procedure (region growing) known to increase the DSC^13^. Fully manual segmentation is reported to provide a lower DSC of 0.68^13^, leaving open the question of whether the region growing algorithm could really provide a much more precise delineation of if the experienced readers were partially influenced by the result of the algorithm. Interesting enough, DSC variability was lower than the variability between experienced readers (0.07 vs 0.13 SD).

During the training of the CNN classifier, the algorithm showed decreased performance in 10 cases of the validation dataset. In each of these cases the AUC was >0.90 but the DSC was zero, suggesting the algorithm managed to identify the tumour tissue in the image but failed to select the correct candidate. Seven cases out of ten were images acquired at the centre B, which applied an imaging protocol with a larger field of view (FOV). The larger FOV inevitably included in the images large chunks of subcutaneous adipose tissue, or anatomical parts – e.g. the testicles –, which were not present in the discovery set (Fig. 4). Although the tumour region resulted in higher probability voxels, after thresholding these adipose tissue or anatomical parts were larger than the tumour and therefore selected as candidate. Including more examples from centre B will likely enable the network to learn to recognize and remove artefacts in these peripheral areas. Figure 4 shows two example cases of a male patients from centre B, where the testicles were misclassified as tumour.

The remaining three cases from centre A showed large fat suppression artefacts, rarely present in the rest of the dataset. Most likely, the scarcity of these examples is the reason of misclassification. This indeed represents the main drawback of supervised learning procedures in general, which are often unable to properly classify underrepresented cases. The same effect can be observed on a microscale in Fig. 1, where the segmentation generated by the algorithm includes non-tumoural hyperintensities, representing some fluid in the rectal lumen. Its under representation in the training set, and the vicinity to the tumour leads the algorithm to assign a tumour probability >0.5 – yet smaller than the probability assigned to the tumour.

Deep learning has been largely used before for segmentation tasks in medical imaging. Out of all possible architectures, we chose this for its straightforwardness and, most importantly, the limited number of images required for training the algorithm. The strategy of using multiple patches from the same patient allows us to generate a large imaging tensors upon which the algorithm can be trained. The patch size chosen allows focusing on a small region without including too much surrounding, but can still generalize textural patterns of specific tissues and organs. Larger patches in fact (e.g. M = 35) as well as smaller patches (e.g. M = 11) resulted in higher training error. These small patches, however, might not provide enough anatomical information needed in some other applications. In Fig. 4, for example, we can see how the algorithm selected a group of voxels outside the pelvis. Fully convoluted end-to-end procedures, such as the one presented in SegNet^34^ or U-Net^35,36^ where 2D slices of MR volumes or entire 3D volumes are fed to a network able to directly generate the target segmentation, would represent an alternative approach worth investigating. Such approach would recognize unlikely tumour locations outside or on the border of the pelvic area, and exclude them automatically. The patch based approach adopted in this study aims to provide the network with an artificially balanced, multiparametric training set from a relatively small dataset via a weighted sampling procedure favouring more challenging regions such as tumour borders and diffusion hyperintensities often found in nearby prostate and seminal vesicles.

Neuroradiology remains the main area of focus of research on segmentation algorithms, and several algorithms and architectures have been proposed for brain tumour segmentation, especially in the context of multiparametric imaging^37–39^. Imaging of the lower abdomen however poses a challenge to automatization, partially because of the bowel movements, which makes it challenging to use voxel-wise mpMRI, and partially because of the high number of artefacts. Although bowel movements can be partially attenuated by deformable registration protocols, such as the one designed in this study, the result is yet suboptimal and needs to be investigated further. Common artefacts are learned as false positive by the algorithm, recognized and removed from the result. Rare artefacts and presence of anatomical parts not seen in the discovery set are still misclassified.

This algorithm represents a preliminary result to support the utilization of deep learning in colorectal MR. We intend to further optimize the protocol, mainly by (1) focusing on alternative architectures which account for anatomical location, and (2) shortening the time needed for the segmentation.

## Conclusions

Our results demonstrate that deep learning can perform accurate localization and segmentation of rectal cancer in MR imaging in the majority of patients. Deep learning technologies have the potential to improve the speed and accuracy of MRI-based rectum segmentations, as manual delineation have shown to be reader dependent and often time consuming, which limits its utility in practice and represents one of the major obstacles in the design of large quantitative imaging studies. Automatic segmentation procedures, such as the one presented in this study, aim to overcome this obstacle by offering a viable alternative to manual delineation. Further validation of these technologies is warranted before clinical application. But if these methods proves reliable its impact in clinical management of rectal cancer could be significant providing an efficient and accurate tool to assess residual tumour burden after preoperative treatment with subsequent better stratification of patients for organ preservation resulting in a higher quality of life.
