# Author Correction: Deep Learning for Fully-Automated Localization and Segmentation of Rectal Cancer on Multiparametric MR

**DOI:** 10.1038/s41598-018-20029-5

**Published:** 2018-02-02

**Authors:** Stefano Trebeschi, Joost J. M. van Griethuysen, Doenja M. J. Lambregts, Max J. Lahaye, Chintan Parmar, Frans C. H. Bakers, Nicky H. G. M. Peters, Regina G. H. Beets-Tan, Hugo J. W. L. Aerts

**Affiliations:** 1 Department of Radiology, the Netherlands Cancer Institute, Amsterdam, The Netherlands; 2 GROW School for Oncology and Developmental Biology, Maastricht University Medical Center, Maastricht, The Netherlands; 3 Department of Radiation Oncology and Radiology, Dana-Farber Cancer Institute, Brigham and Women’s Hospital, Harvard Medical School, Boston, USA; 4 Department of Radiology, Maastricht University Medical Centre, Maastricht, The Netherlands; 5 Department of Radiology, Zuyderland Medical Center, location Heerlen, Heerlen, The Netherlands

Correction to: *Scientific Reports* 10.1038/s41598-017-05728-9, published online 13 July 2017

The original version of this Article contained a typographical error in the spelling of the author Chintan Parmar, which was incorrectly given as Chintan Parmer. This has now been corrected in the PDF and HTML versions of the Article.

